# Systematic Delineation of Media Polarity on COVID-19 Vaccines in Africa: Computational Linguistic Modeling Study

**DOI:** 10.2196/22916

**Published:** 2021-03-16

**Authors:** Sefater Gbashi, Oluwafemi Ayodeji Adebo, Wesley Doorsamy, Patrick Berka Njobeh

**Affiliations:** 1 Faculty of Science University of Johannesburg Johannesburg South Africa; 2 Institute for Intelligent Systems University of Johannesburg Johannesburg South Africa

**Keywords:** COVID-19, coronavirus, vaccine, infodemiology, infoveillance, infodemic, sentiment analysis, natural language processing, media, computation, linguistic, model, communication

## Abstract

**Background:**

The global onset of COVID-19 has resulted in substantial public health and socioeconomic impacts. An immediate medical breakthrough is needed. However, parallel to the emergence of the COVID-19 pandemic is the proliferation of information regarding the pandemic, which, if uncontrolled, cannot only mislead the public but also hinder the concerted efforts of relevant stakeholders in mitigating the effect of this pandemic. It is known that media communications can affect public perception and attitude toward medical treatment, vaccination, or subject matter, particularly when the population has limited knowledge on the subject.

**Objective:**

This study attempts to systematically scrutinize media communications (Google News headlines or snippets and Twitter posts) to understand the prevailing sentiments regarding COVID-19 vaccines in Africa.

**Methods:**

A total of 637 Twitter posts and 569 Google News headlines or descriptions, retrieved between February 2 and May 5, 2020, were analyzed using three standard computational linguistics models (ie, TextBlob, Valence Aware Dictionary and Sentiment Reasoner, and Word2Vec combined with a bidirectional long short-term memory neural network).

**Results:**

Our findings revealed that, contrary to general perceptions, Google News headlines or snippets and Twitter posts within the stated period were generally passive or positive toward COVID-19 vaccines in Africa. It was possible to understand these patterns in light of increasingly sustained efforts by various media and health actors in ensuring the availability of factual information about the pandemic.

**Conclusions:**

This type of analysis could contribute to understanding predominant polarities and associated potential attitudinal inclinations. Such knowledge could be critical in informing relevant public health and media engagement policies.

## Introduction

### COVID-19

COVID-19, a communicable disease caused by SARS-CoV-2, has resulted in the ongoing COVID-19 pandemic [1], with significant health and socioeconomic effects in almost all countries of the world, mainly due to the shutdowns, social distancing, and the resultant business interruptions [2-5]. On March 11, 2020, the World Health Organization (WHO) declared a COVID-19 outbreak due to its global adverse (health and economic) impact [6,7], which is still unfortunately escalating as the world eagerly awaits a medical solution. According to projections by the International Monetary Fund, global economic growth could fall by 0.5% for the year 2020 due to the impact of COVID-19 [7]. A United Nations study revealed that, for the first time since 1900, global poverty could increase [8]. The world unemployment rate has been estimated to hit a mark of 9.4% by the end of 2020, in contrast to the 5.3% seen in 2019 [9]. A forecast by the World Bank projects that Africa is heading toward its first recession in the last 25 years, being driven by the impact of COVID-19 [2]. There is a compelling necessity to achieve an immediate medical breakthrough to curtail the pandemic. Although currently there is no known cure or vaccine for COVID-19, various agencies and governments are working round the clock to proffer much needed solutions. There are indications that some of the vaccines could be ready for clinical trials soon, while others are already undergoing trials.

### Infodemic and COVID-19

Amid the spread of the COVID-19 pandemic and the efforts to provide a solution, there has equally been substantial media interest and widespread misinformation on the web about the scale, origin, diagnosis, prevention, and treatment of the disease [10]. The WHO has expressed particular concern about this *infodemic* and its impact on the fight against the COVID-19 pandemic [11-13]. This “infodemic” may have been facilitated by the global lockdown, isolation, and social distancing situation worldwide, which has led to increased internet and social media use [14,15]. For example, as of May 2020, global internet use was already up by 7% (since the same time in 2019), while the number of social media users increased by 8% (reaching 3.81 billion) since April 2019 [14]. Rozenblum and Bates [16] described the internet and social media as the “perfect storm” with regard to patient-oriented health. Singh [17] called the COVID-19 pandemic–induced media use a “compulsive social media use” and wonders whether it is an addictive behavior. Information diffusion and media coverage can have a positive effect on an epidemic by shortening the duration of the outbreak and reducing its burden [18]; however, if media communication is not properly managed, this can have a negative effect [19,20]. In a study by Brainard and Hunter [21], the authors noted that making 20% of a population unable to share fake advice or reducing the amount of damaging advice spreading online by just 10% can reduce the severity of a disease outbreak.

It is known that medical interventions involving infectious diseases and vaccines in particular are usually predisposed to infodemics [22-24]. Indeed, disease proliferation and associated potential effects take place in a dynamic social space, wherein public knowledge, sentiments, and individual health decisions are influenced by the media and cultural norms [19,25,26]. Studies have shown that an important factor in determining the success of a medical intervention, particularly a vaccination program, is the degree of public acceptance of the proposed intervention [27-29]. Lewandowsky et al [30] observed that the spread of myths regarding vaccinations has led to more parents being reluctant to adopt such measures. The withdrawal of the first US Food and Drug Administration licensed vaccine against the rotavirus in 1999 has been strongly correlated with negative media coverage [31]. Infodemics are known to foster the denial of scientific evidence [32]; incite apathy, cynicism, and extremism [33]; and cause people and institutions to take actions that may not necessarily be in their best interests or for the good of society in general [19,34]. Yu et al [19] observed that, even though medical interventions were able to reduce hepatitis B virus infection by up to 90% among children younger than 5 years using effective and safe hepatitis B (HepB) vaccines, negative media reports about infant death after HepB vaccination resulted in a loss of confidence in vaccines and an approximately 19% decrease in the use of vaccines within the monitored provinces in China. This further emphasizes the importance of individuals’ actions in controlling the spread of the disease and the role of the media and psychosocial effects in this regard, as also observed in the literature [12,13,25,35].

In recognizing the importance of information and corresponding human actions in the management of diseases and other medical emergencies or outbreaks, medical researchers are increasingly exploring the application of computer and mathematical models to analyze communications relating to health problems and to derive value from such resources [18,25,35-37]. One such application is sentiment analysis [38,39]. It is important to understand the prevailing emotions in public media communications, as sentiments could provide a richer set of information about people’s choices and reactions, and in many cases, even determine their decisions [40-42].

### Sentiment Analysis

Sentiment analysis is a multidisciplinary field involved with machine learning (ML) and artificial intelligence (AI), and a subset of natural language processing (NLP) concerned with the systematic extraction, analysis, classification, quantification, and interpretation of affective tonality, opinions, and subjective information in human communications (written or spoken) using computational linguistic methods to derive value of the opinions people express. Sentiment analysis is important because opinions and emotions constitute a critical aspect of humans and play a key role in perceptions of reality, choices, actions, and behaviors [37]. Basic tasks in sentiment analysis include classifying emotional polarity in a communication as positive, negative, or neutral and often scaling such polarities to reflect the depth of the expressed emotions. By understanding prevailing sentiments in communications, health actors can then make more relevant and informed decisions of public relevance and act accordingly to improve the medical experience.

In performing sentiment analysis, language limitations remain a major challenge, as most of the sentiment analysis models and libraries are built in English, which limits their applicability for texts written in other languages. Another problem with traditional sentiment analysis models is the lack of context in labeling lexical items and extracting features, which results in ambiguity in polarity representations. A word or group of words can have different meanings and polarities in different contexts; hence, the global representation of words and sentences could influence the semantics of the words [43]. More contemporary developments in sentiment analysis are shifting toward increasingly context-based sentiment analysis and applicability in other languages [43-45]. Context-aware (ie, context-based) sentiment analysis attempts to tackle the challenge of ambiguity by taking into account all of the text around any given word or words, then processing the logical structure of the sentences, establishing the relations between semantic concepts and assigning logical grammatical roles to the lexical elements to decipher the most relevant meaning of a word or group of words that have more than one definition.

Generally, sentiment analysis models can be largely grouped into two main categories: lexical rule-based techniques and learning-based approaches [43]. The former (ie, lexical rule-based methods) involve the use of predefined lexicons annotated with sentiment polarities, “positive,” “negative,” or “neutral,” which are then used to determine the sentiment of the analyzed text [46]. Lexical rule-based sentiment analyzers such as TextBlob and Valence Aware Dictionary and Sentiment Reasoner (VADER) [47,48], although they use predefined dictionaries, take in to account punctuation such as exclamation marks, emoticons, capitalizations (which gives added intensities to assigned polarities), and negation words (which reverse the polarities). Lexical rule-based sentiment methods have the advantage of being easy to implement because there is no need for the exhausting and arduous task of tagging texts for training. In addition, the approach prevents overfitting because the lexicons are predefined independently of the data being analyzed, a feature that also enables it (lexical rule-based sentiment models) to be used on multiple data sets [46]. On the other hand, the main advantage of (machine) learning-based methods is that they enable the use of domain-specific data sets to train the models, which are then used to analyze a given text. Given relevant and adequate training data sets, this (domain-oriented training) substantially increases the accuracy and level of confidence in text classification and sentiment analysis using learning-based methods. Examples of successful implementation of ML-based sentiment models can be found in the literature [49].

### Applications of Sentiment Analysis

Sentiment analysis has been applied in the fields of medical research, political science, business, mass communication, and education. Specific applications include brand monitoring and reputation management, customer feedback and support, product analysis, market research and competitive research, competitor analysis [50], attitudinal analysis [51], medical records analysis, patient experience analysis, and infodemiology [52-54]. More than ever before, the application of sentiment analysis in the medical field has seen a surge, as both patients and medical practitioners increasingly use web-based platforms such as websites, social media, and blogs to search for treatment-related information and to convey opinions on health matters [55,56]. Melzi et al [57] implemented a supervised learning model to extract sentiments from forums of Spine Health to retrieve patient knowledge. Yang et al [58] demonstrated a sentiment analysis–based framework to derive insights from user-generated content from health social media.

In the context of the ongoing COVID-19 pandemic, Barkur and Vibha [59] performed a sentiment analysis of Indians’ Twitter communications about the nationwide lockdown due to the COVID-19 outbreak. An investigation of the sentiment and public discourse during the pandemic was performed using latent Dirichlet allocation for topic modeling on 1.9 million Tweets written in the English language related to COVID-19 collected from January 23 to March 7, 2020 [60]. Deep long short-term memory models were used to scrutinize the reaction of citizens, public sentiment, and emotions from different cultures about COVID-19 and the subsequent actions taken by different countries [61]. These studies reiterate the usefulness of sentiment analysis in the health domain. Nonetheless, there is no study on the sentiment analysis of media communications regarding COVID-19 vaccines in Africa that can guide health actors to understand the prevailing polarities and to make relevant polices, as the continent awaits the availability of viable vaccines to combat the pandemic.

### Research Justification, Goal, and Questions

Indeed, opinion mining and sentiment analysis in public media is a daunting task because of the large amount of information shared on the internet and social media, particularly in recent times. To give some perspective, Google handles approximately 4.5 million queries every 60 seconds, and approximately 500 million tweets are made every 24 hours [62,63]. In the month of March 2020 alone, about 550 million tweets were made, which included the terms *COVID-19*, *COVID19*, *COVID_19*, *coronavirus*, *corona virus*, or *pandemic*, according to the Pan American Health Organization [64]. Considering the enormity of internet information, researchers are increasingly adopting computer-based algorithms to process, analyze, and interpret such data. This does not only substantially limit the need for human power but also eliminates the associated human biases and inefficiencies in many ways [65,66].

The goal of this paper is to retrieve timely and relevant information that is publicly available regarding COVID-19 vaccines in Africa and interrogate such resources in an attempt to extract the sentiment polarities using computational linguistics models. In this regard, we outlined the following research questions, which constitute the main contributions of this paper:

- What was the media activity patterns regarding COVID-19 vaccines in Africa within the study period of February 2 to May 5, 2020?
- What are the prevailing sentiments (ie, positive, negative, or neutral) in the communications?
- How does the sentiment polarities in Twitter posts compare to those in Google News communications?
- Using three different sentiment analysis approaches, how does the sentiment results from these models compare with each other?
- What were the specific activities or events that might have triggered the prevailing emotions in the communications?

## Methods

This study adopts computational linguistic models (TextBlob, VADER, and Word2Vec–bidirectional long short-term memory [BiLSTM]) to scrutinize communications from popular web-based media sources regarding COVID-19 vaccines in Africa.

### Data Collection and Cleaning

In the collection of data, the following criteria and procedure were used [67].

#### Data Source

Twitter and Google News were selected as the data sources. This is because both media outlets are important web-based information sources for many people. Von Nordheim et al [68] examined the use of different social media platforms (ie, Twitter and Facebook) as journalistic sources in newspapers of three different countries; the authors observed that Twitter is more commonly used as a news source than Facebook, and in comparison to Facebook, Twitter was primarily used as an elite channel [68]. In addition, previous studies have shown that most of the communications on the Twitter platform are truthful, although sometimes it may also be used to propagate false information and rumors [67,69,70]. Moreover, the microblog (ie, Twitter) facilitates the propagation of real-time information to a large group of people, making it an ideal tool for the dissemination of breaking news [69]. Twitter has become a favored communication platform for various social protest movements [71]. On the other hand, Google News is an online media service that aggregates and presents a continuous flow of news content from more than 20,000 publishers worldwide and is available on the web, iOS, and Android. For news in the English language, it covers about 4500 sources. This creates a unique information space, providing a major gateway for consumers to access news, reducing the time and effort needed to regularly check different media sources for updates, and increasing overall user engagement.

#### Query Terms

English language keywords were used to query the data sources instead of hashtags. Specific search terms used were “COVID-19 vaccine Africa,” “COVID19 vaccine Africa,” and “Coronavirus vaccine Africa.” This approach is more inclusive, as it includes communications that have hashtags of the keywords [67]. Moreover, hashtags are often short-lived on the web.

#### Query Period

The sources were queried within the period of February 2 to May 5, 2020, using the search terms. The study period was chosen on the basis of timelines, relevance, and particularly because this was the time frame in which opinions, perspectives, and narratives about the search terms were emerging and could set the tone for subsequent communications as well as make enduring impressions on people’s dispositions.

#### Search Tools

Google News data was retrieved using the Google News application processing interface (API) for Python by Hu [72]. Twitter data were obtained by manually scraping the Twitter web application for publicly available English language posts containing the query terms. This was essentially due to limited resources to subscribe for the Twitter API, which is a paid service. To eliminate any biases, a new Twitter account was used with no previous search history, liked pages, or topics or interests associated with the account.

A total of 569 news headlines and snippets published in English were obtained from Google News and saved as a comma-separated values (CSV) file (Multimedia Appendix 1). Likewise, 637 Twitter posts were extracted and saved in a CSV file (Multimedia Appendix 2) for further analysis. The obtained Twitter and Google News texts were supplied to an algorithm written in Python for data cleaning and “normalization” as described by Sahni et al [73] and Salas-Zárate et al [74] with minor modifications. Briefly, all numbers, special characters, punctuations, and symbols except for commas, periods, exclamation marks, question marks, and apostrophes were removed. Repeated white spaces were replaced with a single space followed by the removal of all stop words. In addition, we autocorrected wrongly spelled English words, removed non-English words, and performed lemmatization. The final cleaned corpus was saved into a Pandas dataframe object for further analysis.

### Opinion Mining and Sentiment Analysis

To provide a more symmetrical and global perspective of the emotive patterns in the data sets, two rule- and lexicon–based ML techniques (ie, TextBlob and VADER) and an advance algorithm, which combines a ML model and a neural network (NN) for NLP, (ie, Word2Vec-BiLSTM) were used to process the cleaned data.

### TextBlob Sentiment Analysis

The TextBlob Python library [48] is an easy-to-use publicly available library for NLP tasks such as sentiment analysis, part-of-speech tagging, classification, noun phrase extraction, translation, tokenization, and n-grams. In this study, we used the NaiveBayes analyzer implementation of TextBlob for sentiment analysis, which is a conditional probabilistic classifier implementation as explained by Hasan et al [75] and Singh et al [76]. The sentiment feature of TextBlob makes use of a predefined dictionary classifying positive and negative words and returns two values, polarity and subjectivity. Polarity is a float between −1 and +1, where −1 means a negative sentiment, +1 means a positive sentiment, and a 0 is considered a neutral sentiment. The subjectivity score is a float between 0 and 1, and represents the degree of personal opinion or judgement rather than factual information. A subjectivity score closer to 0 implies a more objective view, whereas a score closer to 1 represents a more subjective view. The data is generally supplied as a bag-of-words, and after assigning individual scores to each word, the final sentiment is represented by some pooling of all the sentiments. TextBlob has semantic labels that facilitate fine-grained sentiment analysis by recognizing emojis, emoticons, and punctuations such as exclamation marks.

### VADER Sentiment Analysis

The VADER library [47] is a lexicon- and rule–based sentiment analyzer of the English language constructed from a generalizable, valence-based, human-curated gold standard sentiment lexicon and is available with Python’s Natural Language Toolkit library [77]. VADER does not require any training data and is specifically attuned to semantic constructions in social media texts, which are generally informal, yet can be conveniently applied to multiple domains [77,78]. A Python algorithm using the VADER library was developed to unveil and categorize the sentiment in our data set. The output of the analysis is a percentage score of positivity, negativity, and neutrality, as well as a compound score. The compound score is a normalized weighted composite score (ie, normalized to be between –1, most extreme negative, and +1, most extreme positive) that is useful for interpreting the sentiment in a text as a single unidimensional measure of sentiment. On the other hand, the positive, negative, and neutral scores are useful to understand the sentiment in a text in multidimensional measures.

### Word2Vec-BiLSTM Sentiment Analysis

Word2Vec-BiLSTM is an advanced model, which combines ML and AI for NLP. Word2Vec is a ML model that projects natural words, groups of words, or phrases into a multidimensional space, creating vector representations of the word or group of words, which are mapped to vectors of real numbers, otherwise called word embeddings. The resultant two-layer NNs are subsequently trained to reconstruct linguistic contexts of the word or group of words that preserve information about the target word or group of words, the overall context of the word, and the crams variable length sequences to fixed-length vectors. Mikolov et al [79-81] describe in detail the underlying principles and algorithms of Word2Vec. The BiLSTM model [82,83] learns two-way dependencies between time steps of time series data (ie, the output layer gets information from forward and backward states simultaneously), which has presented good results in NLP [84,85]. When given adequate training, Word2Vec-BiLSTM models are highly accurate at capturing the ephemeral qualities of human language and guessing the meanings of words within the context of a sentence. The BiLSTM model and related hyperparameters adopted herein with minor modifications is described by Cherniuk [86]. The training data consisted of 75,000 review entries, with a test size of 0.05% of the training data accordingly labeled as a positive or negative review. The model inputs were feature vectors composed of bigrams and trigrams of the cleaned data set. The model architecture consisted of six layers with a sigmoid activation function, Adam optimizer [87], and binary_crossentropy as the loss function. The model batch size was 100 with an epoch of five. After training, the accuracy of the model was 92%.

## Results

**Figure 1:**
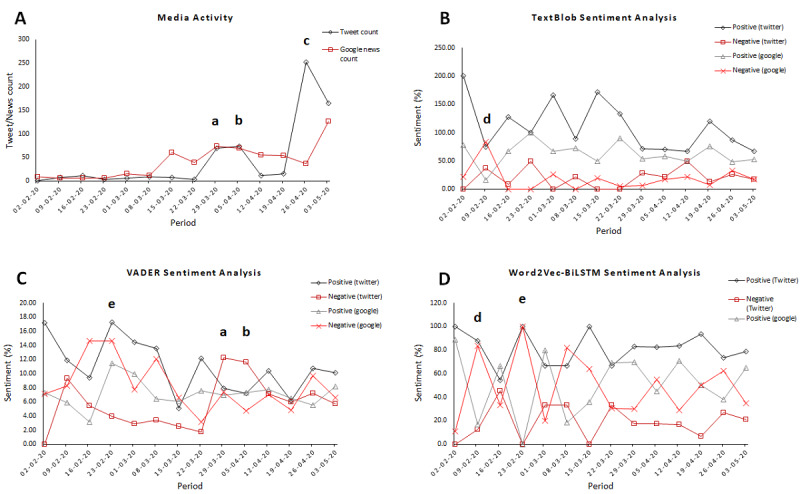
Media activity and sentiment analysis of tweets and Google News headlines and snippets between February 2 and May 5, 2020. (A) Media activity within the study period, (B) TextBlob sentiment analysis, (C) VADER sentiment analysis, and (D) Word2Vec-BiLSTM sentiment analysis. (a) Two French medical doctors made controversial remarks on COVID-19 vaccine testing in Africa on April 1, (b) the World Health Organization strongly remarked about the comments by the French doctors on April 6, (c) Madagascar announced herbal cure to COVID-19 at a virtual meeting of African presidents on April 20, (d) first reported case of COVID-19 in Africa on February 14, and (e) first reported case of COVID-19 in sub-Saharan Africa on February 28. BiLSTM: bidirectional long short-term memory; VADER: Valence Aware Dictionary and Sentiment Reasoner.

The results of media activity within the study period is summarized in Figure 1 [88-95]. There was relatively low activity on Twitter and Google News about the query terms within the first 3 weeks of the study period (ie, between February 2-23, 2020). Google News features on the search topics soared from 39 news items in the eighth week (ie, March 22, 2020) to 73 news features by the ninth week (ie, March 29, 2020). Similarly to the trend on Google News, Twitter posts increased from 3 to 70 between the eighth and ninth weeks of the study period.

Tables 1 and 2 provide a summary of the sentiment polarities in media communications using computational linguistic models. Zooming in on the results for Google News headlines or snippets, it can be observed that average polarities from the VADER analysis revealed that the news was 7% positive, 8% negative, and 85% neutral, with a compound score of –2, which indicates overall negativity in the news tonality. The TextBlob analysis showed that the news was 63% positive, 19% negative, and 22% neutral, with an average subjectivity score of 0.5, indicating a general positivity in the news tonality.

**Table 1:** Sentiment polarities in media communications (Google News headlines or descriptions) analyzed using the TextBlob, VADER, and Word2Vec-BiLSTM models.

Period in 2020	TextBlob					VADER^a^					Word2Vec-BiLSTM^b^	
	Positive	Negative	Neutral	Subjectivity score	Positive		Negative	Neutral	Compound score	Positive		Negative
February 2	77.78	22.22	0.00	0.48	0.07		0.07	0.86	0.02	88.89		11.11
February 9	16.67	83.33	0.00	0.58	0.06		0.08	0.86	–0.01	16.67		83.33
February 16	66.67	0.00	33.33	0.73	0.03		0.15	0.82	–0.35	66.67		33.33
February 23	100.00	0.00	0.00	0.58	0.11		0.15	0.74	–0.11	0.00		100.00
March 1	66.67	26.67	6.67	0.43	0.10		0.08	0.82	0.09	80.00		20.00
March 8	72.73	0.00	27.27	0.64	0.06		0.12	0.82	–0.21	18.18		81.82
March 15	49.18	19.67	31.15	0.40	0.06		0.07	0.87	–0.04	36.07		63.93
March 22	89.74	5.13	5.13	0.37	0.08		0.03	0.89	0.17	69.23		30.77
March 29	53.42	6.85	39.73	0.48	0.07		0.07	0.86	0.02	69.86		30.14
April 5	57.97	17.39	24.64	0.43	0.07		0.05	0.88	0.07	44.93		55.07
April 12	49.09	21.82	29.09	0.34	0.08		0.07	0.85	0.03	70.91		29.09
April 19	75.93	7.41	16.67	0.42	0.06		0.05	0.89	0.10	50.00		50.00
April 26	48.65	32.43	18.92	0.46	0.06		0.10	0.85	–0.14	37.84		62.16
May 3	53.17	17.46	29.37	0.33	0.08		0.07	0.85	0.06	65.08		34.92
Average polarity	62.69	18.60	18.71	0.48	0.07		0.08	0.85	–0.02	51.02		48.98

^a^VADER: Valence Aware Dictionary and Sentiment Reasoner.

^b^BiLSTM: bidirectional long short-term memory.

**Table 2:** Sentiment polarities in media communications (Twitter posts) analyzed using the TextBlob, VADER, and Word2Vec-BiLSTM models.

Period in 2020	TextBlob					VADER^a^					Word2Vec-BiLSTM^b^	
	Positive	Negative	Neutral	Subjectivity score	Positive		Negative	Neutral	Compound score	Positive		Negative
February 2	100.00	0.00	0.00	0.92	0.17		0.00	0.83	0.70	100.00		0.00
February 9	37.50	37.50	25.00	0.34	0.12		0.09	0.79	0.15	87.50		12.50
February 16	63.64	9.09	27.27	0.27	0.09		0.05	0.85	0.12	54.55		45.45
February 23	50.00	50.00	0.00	0.37	0.17		0.04	0.79	0.48	100.00		0.00
March 1	83.33	0.00	16.67	0.59	0.14		0.03	0.83	0.36	66.67		33.33
March 8	44.44	22.22	33.33	0.32	0.14		0.03	0.83	0.38	66.67		33.33
March 15	85.71	0.00	14.29	0.52	0.05		0.02	0.92	0.16	100.00		0.00
March 22	66.67	0.00	33.33	0.40	0.12		0.02	0.86	0.49	66.67		33.33
March 29	38.57	27.14	34.29	0.37	0.08		0.12	0.80	–0.12	82.86		17.14
April 5	36.49	20.27	43.24	0.35	0.07		0.12	0.81	–0.11	82.43		17.57
April 12	33.33	41.67	25.00	0.49	0.10		0.07	0.82	0.10	83.33		16.67
April 19	66.67	6.67	26.67	0.47	0.06		0.06	0.88	–0.02	93.33		6.67
April 26	48.02	23.41	28.57	0.37	0.11		0.07	0.82	0.11	73.41		26.59
May 3	38.18	15.15	46.67	0.32	0.10		0.06	0.84	0.09	78.79		21.21
Average polarity	56.61	18.08	25.31	0.43	0.11		0.06	0.83	0.21	81.16		18.84

^a^VADER: Valence Aware Dictionary and Sentiment Reasoner.

^b^BiLSTM: bidirectional long short-term memory.

Somewhat similar to the TextBlob results, the Word2Vec-BiLSTM analysis unveiled a slightly more positive sentiment in the news communications, as 51% of the news was positive and 49% was negative. On the other hand, taking a look at the results from Twitter, results showed that, for the VADER analysis, the posts were 11% positive, 6% negative, and 83% neutral. The TextBlob analysis showed that the tweets were 57% positive, 18% negative, and 25% neutral, with a subjectivity score of 0.5, indicating an overall positive sentiment in the tweets. Whereas, the Word2Vec-BiLSTM analysis revealed that 81% of the tweets were positive, while 19% were negative, indicating a strong positivity in Twitter communications. Generally, it can be observed that sentiment results from TextBlob were more positive as compared to results from VADER. Elsewhere [96,97], researchers also made similar observations that VADER is more likely to pick up negative tones as compared to TextBlob.

## Discussion

### Principal Findings

Media plays a key role in information dissemination and consumption, which in turn influences people’s opinions, attitudes, and decisions. Herein, we leveraged on the capability of computers to comprehend human language and describe emotional polarities in large amounts of data to delineate the sentiments in Twitter and Google News communications within the period of February 2 to May 5, 2020, regarding COVID-19 vaccines in the continent of Africa. In examining the media activity within the stated period, as expected, it was observed that media activity on the subject matter was relatively low in the first 3 weeks of the study period (ie, between February 2-23, 2020; Figure 1). This is because COVID-19 was just beginning to gain media attention in Africa during that time. In fact, the first case of COVID-19 in Africa was reported on February 14, 2020, in Egypt [93], while the first confirmed case in sub-Saharan Africa, Nigeria to be specific, was reported on February 28, 2020 [94].

It can be observed that there was a spike in media activity on both Twitter and Google News between March 29 and April 5, 2020, with corresponding increases in negative sentiments based on results from TextBlob and VADER (Figure 1 and Tables 1 and 2). This period coincides with negative public reception of the remarks made by two French medical doctors on COVID-19 testing in Africa [89]. The director-general of the WHO, Dr Tedros Adhanom Ghebreyesus, in a press briefing on April 6, 2020, strongly condemned the remarks by the doctors, comments he termed “racist remarks” and ascribed to a “hangover from colonial mentality” [90]. Interestingly coincidental was a public dissent, which culminated in the incineration of a COVID-19 testing facility in Abidjan, capital city of Côte d’Ivoire the same day, on fears that patients with COVID-19 would be treated at the center, because the facility was too close to residential apartments [98,99]. This hostile public response is reminiscent of psychosocial effects during the Ebola outbreaks in Central and West Africa wherein some health workers were attacked on suspicion that they were spreading the disease in their communities, rather than providing medical care [98]. Although the event in Abidjan is seemingly an isolated situation, such experiences reiterate the importance of knowledge and information consumption during the time of a medical emergency or crisis.

Although there was a generally weak decline in positivity from both Twitter and Google News data across the study period, prevailing sentiments were neutral to positive. This gives an indication that generally truer and more evidence-based information regarding COVID-19 vaccines in Africa are circulating on Twitter and Google News as compared to falsehoods. This is because it is expected that, if factual and more science-based information is circulated on the media platforms about the prospects of a viable vaccine to tackle the COVID-19 pandemic in Africa, the communications and corresponding reactions should possess more positivity than negativity. It was observed that there was generally more positivity in Twitter data as compared to Google News data, which might indicate that there was less falsehoods communicated on Twitter as compared to Google News. This trend seems to be in contrast to observations made by other researchers that fake news spreads faster on social media (eg, Twitter, Facebook, Instagram, Snapchat, and WhatsApp) than evidence-based information [100,101]. Notwithstanding, our findings align considerably to those obtained by Pulido et al [67] on public information about COVID-19, wherein the authors noted that for tweets regarding COVID-19, though false information is tweeted more, it had overall less retweets as compared to fact-checking tweets and that science-based evidence tweets captured more engagement than mere facts. Fung et al [24] also made a similar observation, noting that after the declaration of the Ebola outbreak as an emergency in 2014, more accurate information circulated as compared to false information.

These seemingly counterintuitive findings cannot be logically described by chance or normal human behavior alone. Indeed, our observed sentiment patterns seem to be consistent with deliberate and sustained efforts made on various media platforms like Twitter, Facebook, Google, Microsoft, Reddit, and others to limit the spread of misleading and potentially harmful content regarding the COVID-19 pandemic [67,102,103]. For example, in March 2020, Twitter updated its policy guidance to address content sharing on its platform from authoritative sources of global and local health information that goes directly against guidance on COVID-19 [104]. Google, on the other hand, put in place mechanisms to ensure that searches related to COVID-19 on the company’s search engine triggered an “SOS Alert,” with news from mainstream publications including the WHO, National Public Radio, and the US Centers for Disease Control and Prevention displayed prominently [105].

In consonance with efforts made by the public and social media platforms, other national and international entities and health actors have also intensified efforts to ensure accurate and reliable information to the public regarding COVID-19. For example, the WHO’s risk communication team launched a new information platform called WHO Information Network for Epidemics, immediately after it declared COVID-19 a Public Health Emergency of International Concern, devoted to myth-busting, debunking of false information, and sharing of tailored information with specific target groups [13,67]. In response to skepticism regarding COVID-19 vaccines, Media Monitoring Africa, a media organization that aims to promote the development of critical, ethical, and a free and fair media culture in South Africa and the rest of the continent, triggered its Real411 platform, wherein the public can report disinformation regarding COVID-19 to a digital complaints committee [106]. The platform was originally set up during the country’s elections to enable reporting of objectionable speech by members of the public. This strategic media response was deemed necessary following a national survey conducted between April 15-23, 2020, among a representative sample of 600 people regarding COVID-19 vaccines in South Africa, which revealed that 21% of South Africans were strongly unwilling to be vaccinated [106].

### Conclusion

Information constitutes a critical resource for mitigation and curative measures in the fight against the COVID-19 pandemic. This study assessed prevailing affective inclinations in media submissions regarding COVID-19 vaccines in Africa. Contrary to general perception, the results revealed a more passive to positive sentiment, which we could understand within the context of active media policing in ensuring safe and objective information regarding the COVID-19 pandemic. These findings are consistent with previous studies regarding public information about COVID-19. Our analytical approach attempted to provide a more universal and balanced perspective of the emotive patterns in the data sets by adopting three different NLP models (TextBlob, VADER, and Word2Vec-BiLSTM models) with fundamentally different underlying principles and mechanisms. Of course, sentiment analysis is a complex undertaking because speech communications can be highly subjective. For example, certain words or phrases can have different sentiments (ie, positive or negative) depending on the context of the text. Further to that, some narratives such as ironies and sarcasms are extremely difficult for machines and computers to understand because they cannot be interpreted literally. In this regard, we acknowledge the limitations of our approach (including the scope of the search terms and phrases used to query for the data on the internet); nonetheless, we believe that our study provides a reasonable approximation of the media polarity regarding the study topic. In future undertakings, the Word2Vec-BiLSTM and VADER libraries can be trained using more topic-specific vocabulary to improve their overall accuracies and predictability. Ultimately, building on insights from this study, public health and media actors can be stimulated to develop or re-evaluate relevant policies that promote responsible media use and public consumption to maximize the benefits of health interventions amid the COVID-19 crisis. This does not undermine the efficacy of efforts already made to curb misleading information regarding COVID-19 in the public space.

## Appendix

Multimedia Appendix 1 Google News data.

Multimedia Appendix 2 Twitter data.

## Abbreviations

AI: artificial intelligence
API: application processing interface
BiLSTM: bidirectional long short-term memory
CSV: comma-separated values
HepB: hepatitis B
ML: machine learning
NLP: natural language processing
NN: neural network
VADER: Valence Aware Dictionary and Sentiment Reasoner
WHO: World Health Organization
